# East and West

**Published:** 1887-10-01

**Authors:** Leslie Keith

**Affiliations:** Author of "The Chilcotes," "Uncle Bob's Niece," etc.


### East and West.

By Leslie Keith,

Author of "The Chilcotes," "Uncle Bob's Niece," etc.

Chapter I.—The two Miss Franklands.

It was a disputed point in society which of the two Miss Franklands was the more bewitching ; that they were both charming there could not be so much as a doubt in the mind of a sensible person, since they were young, good-looking, and, above all, rich. Before such a combination everyone must surrender. Even if the youth and beauty were lacking, there remains the wealth,—and rich people, as we know, are always delightful. The Miss Franklands were, however, delightful in very different ways. Alicia, the elder, had developed somewhat precociously ; at twenty-four she had plumbed the hollowness of life, and had apparently parted with her last illusion. She was handsome in a stately, Diana-like fashion, with an air of purpose and decision that, taken in conjunction with her caustic speeches, had the effect of keeping aspirants at a respectful distance. She professed herself an advocate for entire frankness and unreserve in the statement of motive, and it must be allowed that she consistently translated her belief into practice. She spoke with an unhesitating directness that was convincing, the more so, probably, because the views and opinions which she ascribed to herself were invariably narrow and chiefly uncharitable. If she had claimed all the virtues there might have been dissenting murmurs even from her devoted followers ; but as it was, society laughed and applauded, and pronounced the elder Miss Frankland to be very piquante. It was noticeable, however, that when she went to balls and dinners and receptions, it was mostly the elders of the other sex who clustered round her ; the grey-beards and veterans—the club-men who were ruefully conscious of being on the wrong side of forty-five—found her cynicism stimulating and amusing ; the younger men, on the other hand, were disconcerted before it, and took their affronts to be healed by Miss Kitty.

"I am very matter-of-fact," Alicia would say, in her clear, incisive tones. "I believe in having a good deal of money, and in getting the utmost possible good out of it for oneself. I suppose there are people who have not much money, and who would like to have more ; I daresay that is rather an unpleasant state of affairs, but it doesn't concern me. The sorrows of humanity never had the least interest for me ; the woes of the poor don't take away my appetite, or rob me of five minutes' sleep. I daresay you are all very much shocked ;" she looked round on her little audience ; "you are all friends and lovers of the destitute, I know. Most of you, I believe, give practical proof of your pity and generosity by subscribing half-a-crown a year to the London charities. I don't. I keep my half-crown, and I add two more to it and buy myself a pair of gloves." She held out a beautifully rounded arm to which the Swedish kid clung in a close embrace, assisted in its task by a bangle that sparkled with diamonds. She contemplated their lustre with calm impartiality, and then looked up and said, with rather a frosty smile, "I am not like you, you see ; I begin and end by being charitable to myself."

Was she right in her self-estimate ? It is at least fair to allow that a man, with his immeasurably superior opportunities of judging, ought to be the best appraiser of his own value : so said wise old Johnson when a man, rescued from death by drowning, presented his deliverer with sixpence. Miss Frankland ticketed herself at something like twopence three farthings (let us not omit the farthings), and the rest of the world at possibly a fraction less. Even Kitty, who shone in the same social firmament, and was voted by a rival band of worshippers a star of greater magnitude, was not allowed to be more noble than the rest of mankind, or permitted to shelter herself behind so much as a shred or tatter of self-delusion.

"You are just as selfish as the rest of us," Alicia would say. "Pray don't suppose I am despising you : selfishness is an inborn instinct ; I should place it among the virtues if I wrote moral tales for young people ; I only beg you not to be so modest as to pretend you don't possess it."

Kitty had so often been told she was selfish that she was able to bear the charge with equanimity, and even with laughter. She was bright and lively, and quite distractingly pretty in a less lofty way than her sister. Do not let it be supposed, however, that she was too frivolous : she was not more frivolous than a young lady who lives in Park Lane has every right to be ; it is only the working girls of the East-end who ought never to laugh, but to be always deeply impressed with the melancholy truth that life is real, life is earnest.

"Of course," Alicia went on, "if it pleases you to represent yourself as generous and sympathetic, I have nothing to say. It isn't true, but half the world soothes itself with the same pleasing delusion. That is how the charity-lists and hospitals get so many half-crowns, and the crossing-sweepers so many pence. After all, a penny, or even thirty pence, isn't an exorbitant sum to pay for an applauding conscience ; you may even call it cheap. If it will add to your comfort, Kitty, you may give two shillings in aid of the Decayed Washerwomen's Fund the next time that very persistent collector calls."

"Thank you," said Kitty, with the usual ripple of smiles, "but it would add more materially to my comfort at this moment if Jack would hold this skein steadily, and allow me to wind it." This allusion to some one of the name of Jack called Miss Frankland's attention to a young man who sat (literally and metaphorically) at Kitty's feet, and she looked at him narrowly. Perhaps his betraying start, and the speed with which his erring hands ceased from hindering another pair of hands busy with the ball, gave a new colour to her thoughts.

"As for you, Jack Fane," she said, in her unemotional tones, "I suppose it amuses you to fancy you are in love ?"

This might have seemed an odd and even an embarrassing thing to say, but for the relations in which the winder and her assistant stood towards each other. It was an understood thing between the Fanes and the Franklands that certain family properties were to be rolled into one by the union, at some unfixed date, of Jack and Kitty ; and the young people, far from resenting this arrangement of their fates, held their parents deserving of credit for desiring to make them happy and more than rich at one stroke. Therefore Jack fired somewhat at Alicia's remark.

"It isn't fancy," he said.

"Ah, if it pleases you to think it is true—"

"It is true," he said, "on my part at least." He looked at Kitty, who had demurely dropped her eyes. "Don't you believe in anything ?" the young man asked, suddenly.

"Not in disagreeable things."

"Love isn't disagreeable." He looked boldly at Kitty this time, and was rewarded by a smile.

"Perhaps not," said Alicia in a tone of well-bred indifference. "I am not emotional or sentimental ; my heart is a very serviceable blood-pumping machine ; that was what it was created for, I believe ; if I were to force it to do some other kind of work it would only bore me."

"No, it wouldn't," he said confidently.

"Oh," she said politely, "if it doesn't bore you, you are quite right to go on making-believe. To be comfortable—not to be bored—that is everything. That is why I consider Lady Ashton such a praiseworthy person; she has discovered or rediscovered a new amusement. I hear papa's knock; I shall go and prepare him to show his gratitude."

"Has she really no heart?" the young man asked, looking after her in dark perplexity.

"She cares for papa," said Kitty with laughing eyes; "I actually got her to admit it once, but she has never forgiven me."

Strangers were, as a rule, rather surprised to hear that there was a papa. They usually discovered the fact at the end of an evening spent in the society of the daughters—at that moment of cloaking when Mr. Frankland emerged from some remote corner and stepped carefully into the brougham behind two sweeping trains. He was usually spoken of after the manner of a by-the-by, a postscript—of a man's letter, it would be pointed out; since it is asserted that a woman leaves the important news for hers. Mr. Frankland was not important, and the main theme was always devoted to his girls. It was thought rather wonderful that he should be the father of two such daughters, and it was also held by some that he should be grateful that they consented to be such a credit to him. For the rest, he was a long, lean, rather lazy man, much given to sitting in easy chairs, and a perfect rake in the matter of cigars. Everybody seemed to understand without being told, that there was no Mrs. Frankland, and it was the general opinion that if her mother had lived Alicia would not have got on with her. No one could speak with authority on the point, however, since Mrs. Frankland had died when her children were mere babies, and Mr. Frankland had never been allowed to replace her. He showed, indeed, no disposition to do so, and entirely acquiesced in the inactive *rôle* appointed him. Possibly, while he played the part of a super he was supported by the humours of the situation. There are consolations in every lot.

If Miss Frankland cared for her father she concealed the fact with tolerable success. When she came sweeping downstairs to the hall, she had somewhat the air of a queen bent on rebuke. He looked up at the lithe and graceful figure, and met it with the modest smile that was so becoming to him.

"You are late," she said.

"Yes," he assented with apology; "I am afraid I am. I was detained at the club. Having nothing to do, I am naturally one of the busiest men in London."

Alicia looked at him with her head a little on one side, in a pose that was graceful. Possibly this young lady inherited some of her peculiar humour from her papa.

"I have found something for you to do this evening," she said.

"That is very kind of you," he answered, with apparent gratitude. "Is it a ball, a reception, a concert, or—a theatre?"

"It is neither of these things; it is something quite new."

Mr. Frankland looked at his daughter with eyes in which there lurked perhaps the slightest suspicion of banter.

"Is it—possibly—a charitable lecture?"

"Papa," said Alicia with an uplifted chin, "you are disagreeable. Did you ever know me to be interested in any charity whatsoever?"

Mr Frankland murmured something to the effect that he certainly never did.

"And do you suppose," she went on, nourishing a sense of injury, "that I would deliberately put myself in a position where I might be made to feel uncomfortable? Of course, I might not feel uncomfortable. I am not a philanthropist; I can listen to the most harrowing tales without being in the least moved, but I should unquestionably feel bored. Your man, or, worse still, your woman-lecturer, with an appeal compounded of pathos and horrors and a collection to finish up with, would certainly bore me It would bore you too," she challenged him.

He admitted that it might.

"You are going with Kitty and me to Lady Ashton's soap-bubble party."

"To her—?" he questioned politely.

"To blow soap-bells," said Alicia, in her clearest tones. "The whole credit of this delightful invention is due to Lady Ashton. I think"—she lifted her eyebrows—"we have exhausted everything else."

"It certainly," said Mr. Frankland with due gravity, "does not demand a too great intellectual effort."

"No," assented Alicia, looking him straight in the eyes, "it does not; it is quite simple, and extremely innocent. I have no doubt it will interest you very much. It is better for you than staying at home and smoking cigars."

"Having thus admirably provided for her father's amusement, Alicia went upstairs to ponder over a toilet suitable to the occasion. It was one of the very few pleasures left in a hollow or disappointing world that she was able to dress a little better than the womenkind of her acquaintance.

London society is still open to the charge made of the Grecian city long ago, and when Lady Ashton had her happy inspiration, all the Athenians of her circle, and many of those who did not properly belong to it, flocked to her handsome house in Grosvenor Square. There are no Athenians in Whitechapel or Mile End, and perhaps it is just as well, since, however much they might long for something new, they would not be in the least likely to get it. They very properly recognise, down there, that it is only the genteel circles of Belgravia and Mayfair whose constitutions demand perpetual amusement.

Lady Ashton's entertainment was a brilliant success, and the society journals duly gave it their meed of praise. A real prince was present, and lords were plentiful, and they showed a most moving zeal in this return to the pastimes of their childish days. "It was this rebound to youth and innocence," cried society by the mouth of its organs, "that was so cheering, so hopeful a sign of the times. To witness a grave and reverend senior, whose eloquence had shaken the House of Lords, thus unbending, and artlessly reverting to the joys of his pinafore days, was enough to bring tears to the eyes of the most hardened." Mr. Frankland quite agreed with this sentiment when he read it on the following Saturday in *Mayfair*, though he possibly did not interpret it in the manner that the paragraphist intended. But, then, Mr. Frankland did not love a pipe unless there were tobacco in it, and to be offered soapy water as a substitute may have soured him. He took his usual modest position against the supporting angle of a wall, and become a spectator of the scene.

The pipes, of a blameless white, were handed about by footmen, who bore them solemnly on silver salvers; the soapy water was distributed in bowls of the rarest old china. Some of the ladies wore costumes which strove to rival the evanescent tints of the bells that floated about for a brief moment of glory before vanishing, and of these the shimmering draperies out of which Alicia's long neck rose like a flower-stem were pronounced to be the most successful.

Kitty's laughing lips could not steady themselves for sufficiently long together to make her a proficient in the new play, but Alicia covered herself with glory by the ease and dexterity of her art. She was very earnest over it; as serious as if the chief end of life were the manufacture of soap-bells, and the gentlemen who aspired to her notice found that to win it they had to be serious too. So that a great deal of real business went on in her corner, and the manufacture flourished there as it did not elsewhere, where the soap-blowers yielded to the temptation to talk, whisper, laugh, exclaim, or flirt; and when Alicia got back to her room at night, she was able to assure her reflection in the glass that the evening had been profitably spent, since she had not been bored.

So far, Alicia had managed extremely well. For a philosopher who has found the world a sham, to have lived in it twenty-four years without excessive boredom is something, and as yet she had encountered no trouble that was not perfectly well-dressed and well-bred. But even she could not always hope to be successful, or to adjust the world to meet her claims on it. While she was striving with an energy that might almost be called heroic to fill up all the blanks and pauses in her day with occupations that should amuse and distract, there was advancing from the North a power that was to shatter her confidence, and prove to her that she could, no more than the rest of us, refuse her share of the common lot, or shelter herself behind the barrier of her private objections from the obligations life lays on us all.

At the present moment, indeed, it was a little difficult to conceive of Miss Frankland as a traveller on the common highway, where other toiling wayfarers might jostle, and hustle, and possibly bruise her. It was easier to sustain a vision of her driving through life in a carriage and pair, on a by-path specially constructed for herself, where naught should alarm or annoy, or hinder her royal progress. But Fate, which no man can withstand, had decreed otherwise.

(*To be continued.*)

